# DPPH-Scavenging and Antimicrobial Activities of Asteraceae Medicinal Plants on Uropathogenic Bacteria

**DOI:** 10.1155/2020/7807026

**Published:** 2020-05-14

**Authors:** Phan-Canh Trinh, Le-Thi-Thanh Thao, Hoang-Tran-Viet Ha, TuAnh Nguyen

**Affiliations:** Department of Microbiology and Parasitology, Faculty of Pharmacy, University of Medicine and Pharmacy at Ho Chi Minh City, Ho Chi Minh 700000, Vietnam

## Abstract

Asteraceae species were widely applied in traditional medicines in Asian countries as sources of natural antioxidants and antimicrobial agents. This study aimed to evaluate DPPH-scavenging capacities and antimicrobial activities of nine Asteraceae species collected from Southern Vietnam. Antioxidant and antimicrobial activities were determined by standard protocols. Essential oils from *Ageratum conyzoides, Helianthus annuus,* and *Artemisia vulgaris* indicated significant inhibitory effects on *Staphylococcus aureus* and *Candida* spp. Crude extracts and fractions from *Taraxacum officinale, Chrysanthemum morifolium*, *A. conyzoides,* and *Tagetes erecta* showed inhibitory ability on at least one testing bacterial strains including *S. aureus*, *Escherichia coli*, *Klebsiella pneumoniae*, and *Pseudomonas aeruginosa*. In a study on clinical isolates, ethyl acetate fraction from *A. conyzoides* flower displayed the most potent effect on uropathogenic *E. coli* and *K. pneumoniae* with MIC at 1.25–10 mg/ml and 5–12.5 mg/ml, respectively. DPPH-scavenging assay indicated that *T. erecta* extract had the lowest IC_50_ (17.280 *μ*g/ml) and is 2.4 times higher than vitamin C (7.321 *μ*g/ml). This study revealed that *A. conyzoides* has good potential against uropathogenic *E. coli* and *K. pneumoniae,* and therefore could be applied for prophylactic treatment of urinary infection.

## 1. Introduction

In recent years, antibiotic resistance has become more sophisticated, putting mankind into the postantibiotic era. Many clinical Enterobacteriaceae strains such as *Escherichia coli* and *Klebsiella pneumoniae* have extended-spectrum beta-lactamase (ESBL) and carbapenem-resistant Enterobacteriaceae (CRE) [1–3]. Polymyxin plasmid-mediated resistance gene (mcr-1) especially was observed in *E. coli* strains, which was isolated from animals and in patients with infection during 2011–2014, in China [4]. Moreover, it is with profound concern that mcr-1 could be transferred to *K. pneumoniae* and *Pseudomonas aeruginosa* via transformation [4]. In 2016, the first report about mcr-1 gene in a patient with urinary tract infections (UTIs) in Pennsylvania, the United States, was shown by Abbasi [5]. According to recent reports, the causative agents of UTIs include uropathogenic *E. coli, Klebsiella pneumoniae, Enterococcus* spp., *Staphylococcus saprophyticus*, group B *Streptococcus* (GBS), *Proteus mirabilis, Pseudomonas aeruginosa, Staphylococcus aureus,* and *Candida* spp. [6–10], in which *Escherichia coli* is the most common causative agents of both uncomplicated and complicated UTIs [6].

Herbal extracts and essential oils were used as foods such as floral beverages, functional foods, and traditional medicines in many years, with minimal known “side effects” on human health. Using herbal remedies might help in reducing dependence on antibiotic therapies and minimizing antibiotic resistance [11].

The Asteraceae family (Compositae) is a widespread family of flowering plants, including 32,913 species names, belonging to 1,911 plant genera, distributed in 13 subfamilies [12]. The tropics, subtropics, and temperate regions are the natural habitats of Asteraceae species [13]. They usually contain a large amount of essential oil, polyphenols, and flavonoid compounds, which are often studied for antimicrobial and antioxidant activities [14–19].

Although there were many reports for antimicrobial and antioxidant effects of Asteraceae species, applications of these extracts in treating infectious diseases need an evaluation of pathogenic bacterial strains isolated from clinical specimens [11]. In the study, we screened antimicrobial and antioxidant activities of ethanol extracts and essential oils from nine species of Asteraceae on 30 clinical strains causing urinary tract infection, collected from District 2 Hospital, Ho Chi Minh City, Vietnam. The target was seeking the best extract to apply for a healthcare serum to prevent recurrent UTIs. The antioxidant activity might be a protective factor for urinary tract epithelium to avoid the impact of oxidative stress.

## 2. Materials and Methods

**Figure 1:**
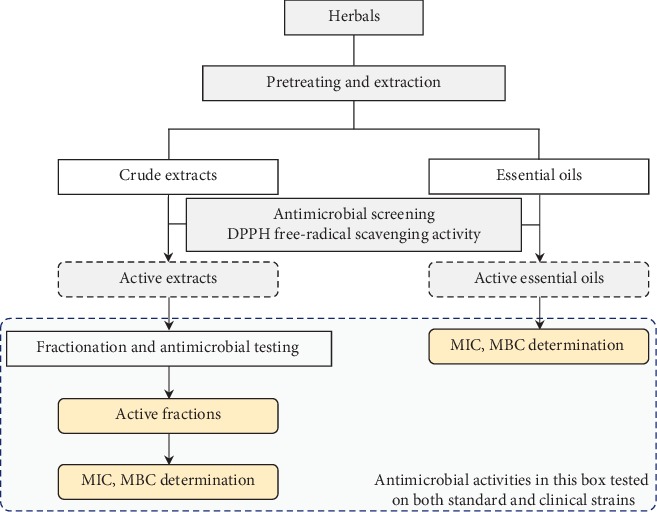
Schematic representation of the experimental layout.

We conducted investigations on antimicrobial activities of nine Asteraceae species collected from Southern Vietnam, following Figure 1. In particular, after pretreating and extracting herbal samples to obtain crude extracts and essential oils, we evaluated the antimicrobial effect by applying the diffusion method. The extracts which show activity were fractionated by *n*-hexane, chloroform, and ethyl acetate, respectively. The well-agar-diffusion method was used to determine the antimicrobial capacities of fractions. Fractions which indicated inhibitory zone were evaluated with MIC and MBC. DPPH free-radical scavenging assays were carried out on crude extracts [20, 21].

### 2.1. Plant Authentication and Preparation

Asteraceae-plant samples were collected from Southern Vietnam from March to May 2016. These samples were identified at the Botany Department, Faculty of Pharmacy, University of Medicine and Pharmacy at Ho Chi Minh City. These species are usually used as traditional medicines in Vietnam.  Table 1 shows common names and general uses following the botanical nomenclature of nine species used in this study.

After harvesting, the samples were washed under running water to remove dust and rinse with distilled water to drain. Subsequently, they were dried in the shade, and afterwards the dried plant materials were finely grounded by mechanical grinders. The powder was stored in tightly closed glass containers in the dark at room temperature.

### 2.2. Preparation of Plant Extracts

#### 2.2.1. Preparation of Essential Oils

Plant samples were cut into small pieces after washing under running water. Plant materials (100 g) were placed in a flask (1 L) together with distilled water. Clevenger apparatus was used for distillation of essential oils. After steam distillation (about 3 hours), the oil was isolated and dried over anhydrous sodium sulfate (Merck). The essential oils were used directly for antimicrobial assay.

#### 2.2.2. Ethanol Crude Extracts

Plant materials (50 g) were extracted by cold soaking with 500 ml of 96% ethanol (Xilong Chemical) for 24 hours at 10 : 1 solvent-to-sample ratio (v/w). Then, the mixtures were filtered through Whatman filter paper. The extracts were allowed to evaporate at a temperature of 45–50°C with water bath WNB 29 (Memmert). These steps were repeated three times to achieve maximal extraction of compounds. Dried crude extracts were weighed and kept at −35°C till further use (not more than one month). These extracts had been screened for antimicrobial activity with well agar diffusion method to choose the good antimicrobial extracts for the next step.

#### 2.2.3. Fractionation of the Ethanol Crude Extracts

Ethanol crude extracts, which possess the strong antimicrobial effect, were subjected to liquid/liquid extraction with *n-*hexane, chloroform, and ethyl acetate, respectively. After evaporating solvent, the antimicrobial activity was determined for each fraction.

Stock solutions of crude extracts and fractions were prepared at a concentration of 100 mg/ml in 10% dimethyl sulfoxide (DMSO, Merck).

### 2.3. Preliminary Phytochemical Screening

The ethanol crude extracts were analyzed for phytochemical constituents for the identification of various classes of compounds, according to Maria et al. [32].

### 2.4. Microorganism Strains and Culture Conditions

Microbial strains from American Type Culture Collection (ATCC) were used in this study for preliminary antimicrobial assays, which included methicillin-sensitive *Staphylococcus aureus* ATCC 25923 (SA), methicillin-resistant *S. aureus* ATCC 33591 (SR), *Enterococcus faecalis* ATCC 29212 (EF), *Escherichia coli* ATCC 25922 (EC), *Klebsiella pneumoniae* ATCC 35657 (KP), *Pseudomonas aeruginosa* ATCC 27853 (PA), and *Candida albicans* ATCC 10231 (CA). Two clinical *non-albicans* strains, *Candida glabrata* ND31 (CG) and *Candida tropicalis* PNT20 (CT), which were provided by Anh et al. [33] and clinical isolates (15 *E. coli* and 15 *K. pneumoniae),* which were isolated from District 2 Hospital, Ho Chi Minh City, Vietnam, in 2016, were also applied for antimicrobial investigation on the potential extracts.

These strains were preserved in 25% glycerol at −80°C. One strain tube was thawed rapidly at 37°C and cultured in 10 ml Brain heart infusion (BHI, Merck) at 37°C over 24 hours. The bacteria were streaked on BHI agar (BHA, Merck) at 37°C over 24 hours. One to five colonies were used to prepare bacterial suspension to match a 0.5 McFarland standard (1–1.5 × 10^8^ CFU/ml). Mueller-Hinton agar medium (MHA, Merck) and MHA supplied 2% glucose medium (MHGA, Merck) were used for determination antibacterial and antifungal activity, respectively.

### 2.5. Antimicrobial Diffusion Method

The antimicrobial activity of the samples was initially evaluated by the well agar diffusion assay for the extracts and disc diffusion assay for the essential oils [21]. The growth medium was poured into Petri dishes at 45–50°C, approximately 4 mm depth, and they were left to solidify in the laminar-flow hood. Subsequently, a sterile cotton swab was dipped into overnight microbial suspensions (adjusted to a turbidity of 0.5 McFarland standard). Agar plates were inoculated by evenly streaking cotton swab over the agar medium.

As for extracts, wells with a diameter of 6 mm were cut in the inoculated-agar medium with a sterile cork borer. Stock solutions of the samples were diluted in sterile distilled water to get 100 mg/ml concentration. The tested samples and controls (50 *μ*l) were dispensed into the wells.

As for essential oils, 20 *μ*l of the oils was applied on filter paper discs (6 mm). These discs were put on the inoculated-agar surface.

The plates were incubated at 37°C for 24–48 hours. After that, the diameters of growth inhibition zones were measured by the electronic vernier caliper (Insize 1112–200). The following control agents were used: positive control agents, ampicillin 20 *μ*g/ml (for bacteria) and ketoconazole 20 *μ*g/ml (for yeasts); negative control agent, 10% DMSO.

### 2.6. Determination of Minimum Inhibitory Concentration

Determination of minimum inhibitory concentrations (MIC) of the extracts and essential oils was done using the agar dilution method [21]. Stock solutions were diluted with melted agar to a concentration range so that the following concentration is equal to half the previous concentration. Subsequently, the agar was poured into Petri discs and waited for them to solidify in the laminar-flow hood. Microorganism suspensions at 0.5 McFarland were diluted by 0.85% NaCl solution to reach 1–1.5 × 10^7^ CFU/ml for bacteria and 1–5 × 10^6^ CFU/ml for *Candida* spp. These suspensions were spotted (1 *μ*l) on the agar surface. Bacterial or yeast colonies growth at the spot after incubating at 37°C for 24–48 hours indicated for the assay. The MIC is the lowest concentration of antimicrobial agent that completely inhibits growth. The experiment was replicated three times.

### 2.7. Determination of Minimum Bactericidal Concentration

To determine the minimal bactericidal concentration (MBC), the spots at MIC, 2MIC, 4MIC, and 8MIC were washed with 1 ml of 0.85% NaCl. The 100 *μ*l of the washing suspension was spread evenly over BHA agar. After 24–48 hours of incubation at 37°C, the number of surviving bacteria was determined. The MBC was defined as the lowest extract concentration at which 99.9% of the bacteria have been killed. The experiment was replicated three times.

### 2.8. DPPH Free-Radical Scavenging Activity Assay

The 0.25 mg/ml 2,2-diphenyl-1-picryl-hydrazyl-hydrate (DPPH, Sigma Aldrich) solution in methanol (working solution) was used to determine the antioxidant capacity. Stock solutions were prepared at the sample concentration of 10 mg/ml. The preliminary tests were carried out on TLC Silica gel 60 F₂₅₄ (Merck). After impregnating 2 *μ*l/spot of stock solutions on the TLC plate, the plate was dipped into the DPPH working solution and incubated at the room temperature 30 minutes. The positive test indicated the yellow on the violet background [34].

Reaction mixtures consisted of stock solution, 2 ml DPPH working solution, and methanol as a solvent to have a sample concentration from 0.1 to 0.5 mg/ml. The negative controls had only the solvent instead of the testing solution. The mixtures were incubated for 30 minutes at 37°C in the dark. The decrease in the absorbance at 517 nm was measured (*A*_*E*_) [34]. The experiment was carried out in triplicate. Samples and positive control ascorbic acid were tested in triplicate over the same range of sample concentrations. Radical scavenging activity was calculated using the following formula:

(1) $$\begin{array}{c} \text{SC}\%=100\%x\frac{\left(A_{B}-A_{E}\right)}{A_{B}}\left(\text{\%}\right), \end{array}$$

where *A*_*B*_ is absorbance of the blank sample and *A*_*E*_ is absorbance of the plant extract.

The antioxidant activity was expressed as the IC_50_ value. This value was determined from the plotted graphs of scavenging activity against the concentration of the sample.

## 3. Results and Discussion

### 3.1. Results

#### 3.1.1. Preliminary Phytochemical Screening

Table 2 depicts various classes of phytoconstituents presenting in testing herbs. In general, tannins, flavonoids, and phenolics were found in all testing herbs. *A. conyzoides*, *H. annuus,* and *A. vulgaris* possess a large amount of essential oil.

#### 3.1.2. Antimicrobial Screening Assay

Among ethanol crude extracts tested, there were four plant species including *Ageratum conyzoides*, *Chrysanthemum morifolium*, *Tagetes erecta*, and *Taraxacum officinale* indicating the antibacterial activities from one to four standard bacterial strains. None of them had the anti-yeast activity on three yeast strains listed in Table 3. To be more specific, *A. conyzoides* and *T. erecta* expressed the broad-spectrum antimicrobial effect on Gram-positive (20–22 mm on MSSA and MRSA) and Gram-negative bacteria (11–18 mm) while simultaneously *T. erecta* manifested the inhibitory capacity on *P. aeruginosa* (13 mm). Besides, *C. morifolium* and *T. officinale* indicated the activity only on methicillin-sensitive *S. aureus*.

The ethanol crude extracts, which showed antimicrobial activity (active extracts), were decanted with different polarization solvents including *n*-hexane, chloroform, and ethyl acetate to obtain fractions. The diffusion method was conducted to determine antimicrobial activities of these fractions on microbial strains. Noticeably, antimicrobial agents are usually distributed in moderate polarity fractions such as chloroform and ethyl acetate (Table 4). The ethyl acetate fraction of *A. conyzoides* displayed the IZD on MSSA (23 mm), MRSA (21 mm), *K. pneumoniae* (15 mm), and *E. coli* (14 mm). Similarly, *T. erecta's* fraction of ethyl acetate shows IZD from 11 to 18 mm on MSSA, MRSA, *K. pneumoniae,* and *P. aeruginosa*.

The essential oils of *A. conyzoides* (aerial parts)*, A. vulgaris* (aerial parts), and *H. annuus* (flowers) demonstrated antimicrobial activities (Table 5). Although ethanol crude extracts of *A. vulgaris* and *H. annuus* do not have inhibitory capacity on testing microorganisms, this test revealed the effect of their essential oils on *S. aureus* and *Candida* spp.

MIC and MBC values of crude extracts and fractions from the four selected plant parts *(Ageratum conyzoides*, *Chrysanthemum morifolium*, *Tagetes erecta*, and *Taraxacum officinale)* were determined (Table 6).

MIC and MBC values of the three essential oils, *A. conyzoides, A. vulgaris,* and *H. annuus,* were shown in Table 7.

Through these results, we found effects of extracts from *A. conyzoides* and *T. erecta* against *E. coli* and *K. pneumoniae* being two leading infectious agents in UTIs. In order to explore the best extract to prevent recurrence UTIs, we evaluated the crude extracts and fractions of *A. conyzoides* and *T. erecta* on 15 *E. coli* and 15 *K. pneumoniae* isolates from urine specimens at District 2 Hospital, Ho Chi Minh City.

The well agar diffusion assay was used for analyzing the activity of the investigated extracts. The results showed that only the ethyl acetate fraction from *A. conyzoides* had antibacterial activity against tested isolates. MIC values of the ethyl acetate fraction of *A. conyzoides* were determined by the agar dilution assay on the isolates of *E. coli* and *K. pneumoniae* that had minimum and maximum diameters of growth inhibition zone and on ESBL-producing isolates. The width of inhibition zones and minimum inhibitory concentration (MIC) is shown in Table 8. In detail, the IZD on *E. coli* and *K. pneumoniae* is indicated from 10.83 mm (E3) to 23.72 mm (E72) and from 9.33 mm (K18) to 19.73 mm (K17), respectively. *A. conyzoides'*s ethyl acetate fraction displayed MIC values on E3, E72, K17, and K18 being 12.5, 5, 10, and 1.25 mg/ml, respectively. ESBL-producing strains, E68, and K26 expressed MIC 6.25 and 2.5 mg/ml, respectively.

#### 3.1.3. Free Radical Scavenging Activity

**Figure 2:**
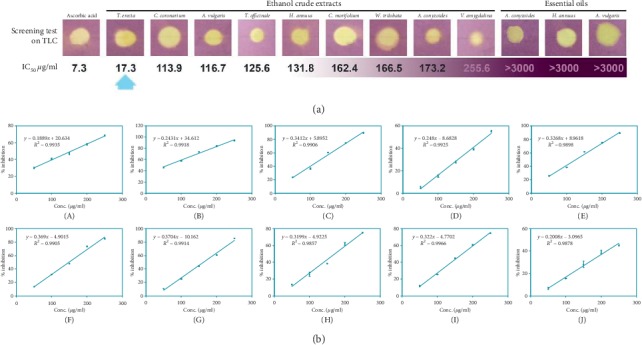
(a) DPPH screening test on TLC and IC_50_ values. (b) The plotted graphs of scavenging activity against the concentration of the ethanol crude extracts and ascorbic acid. A. Ascorbic acid. B. *T. erecta*. C. *C. coronarium*. D. *A. vulgaris*. E. *T. officinale*. F. *H. annuus*. G. *C. morifolium*. H. *W. trilobata*. I. *A. conyzoides*. J. *V. amygdalina*.

DPPH screening test showed the good antioxidant effects of all crude extracts and essential oils. Free-radical scavenging activity of total ethanol extracts was quantitatively determined using a DPPH assay. IC_50_ value represents the concentration of tested extract, at which the inhibition of test activity reached 50% (Figure 2). The results were graphed by Microsoft Excel 2016. The IC_50_ values of essential oils were too large (3000–6000 *μ*g/ml), which were outside the linear range.

The crude extract of *T. erecta* flower showed the significant scavenging effect for free radicals with IC_50_ = 17.3 *μ*g/ml, 2.4 folds comparing to ascorbic acid.

### 3.2. Discussion

Among the tested extracts and essential oils, there were four crude extracts (*Ageratum conyzoides*, *Chrysanthemum morifolium*, *Tagetes erecta*, and *Taraxacum officinale)* and three essential oils (*A. conyzoides, A. vulgaris,* and *H. annuus*) indicating the antimicrobial activity against nine bacteria and three yeast strains. While the crude extracts only had effects on bacteria, the essential oils had effects on both bacteria and yeast strains. There are many reports about antiseptic, antimicrobial, antioxidant, and insecticidal activities of essential oils [35]. Chemical constituents represented in essential oils are usually derived from terpenes, phenolic compounds, and aromatic or aliphatic acid esters, which can partition into the lipids of bacterial and mitochondrial membrane resulting in disturbing the cell structures. The death of cells is caused by leakage of a large number of essential molecules and ions from the bacterial cell [36].

In this research, the ethanol crude extract from *Tagetes erecta* flower showed the highest inhibitory effect on MSSA (0.78 mg/ml), MRSA (3.13 mg/ml), *K. pneumoniae* (1.25 mg/ml), and *P. aeruginosa* (2.5 mg/ml), in comparison with other extracts. Recently, many reports demonstrated that the extracts of *Tagetes erecta* inhibited *S. aureus*, *P. aeruginosa*, *K. pneumoniae*, and *P. mirabilis* [37–39]. Padalia and Chanda (2015) reported MIC values on those bacteria in the range of 62–1250 *μ*g/ml [37].

*Ageratum conyzoides* aerial part extracts had an effect on MSSA, MRSA, *K. pneumoniae,* and *E. coli,* but the MIC values were higher than *T. erecta* extracts. Amadi et al. demonstrated that ethanol extract of *A. conyzoides* displayed the significant inhibitory zone on *Streptococcus mutans* [40]. Following the report of Akinyemi et al. (2005), ethanol extract from *A. conyzoides* indicated MIC and MBC on MRSA of 43 *μ*g/ml and 63.2 *μ*g/ml, respectively [41]. Kouame et al. studied essential oils extracted from flower and stem of *A. conyzoides* [42]; the results displayed antibacterial activities with MIC in the range of 64 to 256 *μ*g/ml on both Gram-positive and Gram-negative bacteria [42]. In this study, the MIC values were higher than the previous reports [41, 42]; these differences can relate to the dissimilarity of the time of sampling and extraction condition.

Ethyl acetate fractions (EA) from *A. conyzoides*, *T. erecta*, *C. morifolium*, and *T. officinale* showed activities against *S. aureus*. It is worth noting that EA from *A. conyzoides* and *T. erecta* displayed considerable impacts on Gram-negative bacteria including *E. coli*, *K. pneumoniae* (for *A. conyzoides*), and *P. aeruginosa* (for *T. erecta*). However, evaluating these EA on uropathogenic isolates revealed that merely the EA of *Ageratum conyzoides* expressed the capacity against uropathogenic isolates. Particularly, it was active against *E. coli* and *K. pneumoniae* producing ESBL, the strains that were highly resistant in clinical infection. Hence, *Ageratum conyzoides* is a good candidate for antiuropathogenic bacteria.

*A. conyzoides, A. vulgaris,* and *H. annuus* essential oils expressed antimicrobial activities against both bacteria (MSSA and MRSA) and yeasts (*C. albicans, C. glabrata,* and *C. tropicalis*). Our investigations witnessed anti-MRSA and anti-*Candida* effects of *A. vulgaris* essential oil. Those capacities could be attributed to a large amount of mono- and sesquiterpenes compounds such as sabinene, *β*-thujone, chrysanthenone, camphor, and borneol in *A. vulgaris* oil [43].

DPPH is a free radical, stable at room temperature, which produces a violet solution in methanol. It is reduced in the presence of antioxidant molecules, making the color of the solution turned yellow. The use of DPPH provides an easy and rapid way to evaluate antioxidants.

All nine ethanol extracts were capable of capturing DPPH free radicals. *T. erecta* extracts had the lowest IC_50_ value, 17.3 *μ*g/ml, which is only 2.4 times higher than the IC_50_ value of ascorbic acid. This result could be due to the presence of a large amount of flavonoid and phenolic compounds in *T. erecta* as mentioned in preliminary phytochemicals. In the previous studies, marigolds displayed to contain quercetagetin, glucoside of quercetagetin, phenolics, syringic acid, methyl 3,5-dihydroxy-4-methoxy benzoate, quercetin, thienyl, and ethyl gallate [44]. Quercetin and ethyl gallate are potent antioxidant compounds in both *in vitro* and *in vivo* [45–47]. Containing many compounds with strong free-radical scavenging effects of *T. erecta* showed the ability for treatment of diseases caused by free radicals such as cancer, diabetes, and cardiovascular. However, this capacity needs to be evaluated more accurately by *in vivo* tests.

## 4. Conclusions

The fraction of ethyl acetate extracted from *A. conyzoides* possesses antimicrobial activities on uropathogenic *E. coli* and *K. pneumoniae* collected from District 2 Hospital, Ho Chi Minh City, Vietnam. This fraction is the potential to apply for healthcare serum in prophylactic recurrence UTIs. *T. erecta* showed the highest potent in DPPH radical scavenging assay, which could become a good candidate for the antioxidative agent in food and cosmetic products.

## Figures and Tables

**Table 1:** The investigated plants.

Nomenclature	Common name	Traditional uses^*∗*^	Part tested^†^
*Ageratum conyzoides* L.	Billygoat weed	Sinusitis, anti-inflammation [22]	Aerial parts, essential oils [22]
*Artemisia vulgaris* L.	Mugwort	Skin ailments, wounds, ulcers [23]	Aerial parts, essential oils [23]
*Chrysanthemum coronarium* L.	Crown daisy	Pain relief, fever, dysentery [24, 25]	Aerial parts [25]
*Chrysanthemum morifolium* Ramat.	Florists chrysanthemum	Pimples, dermatitis, fevers [26]	Flowers [26]
*Helianthus annuus* L.	Sunflower	Anti-inflammatory, malaria [27]	Flowers, essential oils [27]
*Tagetes erecta* L.	Mexican marigold	Dysentery, asthma, ulcer [28]	Flowers [28]
*Taraxacum officinale* F. H. Wigg.	Dandelion	Hepatitis, bronchitis, pneumonia [29]	Aerial parts [29]
*Vernonia amygdalina* Del.	Bitter leaf	Fever, measles, parasites [30]	Leaves [30]
*Wedelia trilobata* L.	Wedelia	Fever, infection [31]	Aerial parts [31]

^*∗*^In this column, there are presented only such traditional uses which can imply the presence of antimicrobial compounds. ^†^ Part tested: the part of the plant used in this study.

**Table 2:** Phytochemical profile of ethanol crude extracts.

Group of compounds	TO	CM	AC	CC	HA	VA	AV	TE	WT
Carotenoid	−	−	−	−	−	−	−	−	−
Free triterpenoids	+	−	−	−	−	−	−	+	−
Alkaloids	−	−	−	−	+	+	+	+	−
Coumarins	−	−	−	−	−	−	−	−	−
Anthraglycosides	+	−	−	−	−	+	−	−	−
Flavonoids	++	+++	++	+	++	+	+	+++	+
Heart glycolysis	−	−	−	−	−	−	−	−	−
Tannins	+++	++	+++	+	++	++	++	+++	++
Phenolics	+++	+++	+++	++	++	++	++	+++	+
Saponins	+	+	−	+	−	+	+	+	+
Organic acids	−	−	−	−	−	−	−	−	−
Reducing agent	+	+	+	+	+	+	+	+	+

TO: *T. officinale*; CM: *C. morifolium*; AC: *A. conyzoides*; CC: *C. coronarium*; HA: *H. annuus*; VA: *V. amygdalina*; AV: *A. vulgaris*; TE: *T. erecta*; WT: *W. trilobata*.

**Table 3:** The inhibitory zone diameter (IZD, mm) of the ethanol crude plant extracts determined by agar well diffusion assay.

Plant species	Inhibitory zone diameter (mm)								
	SA	SR	EF	KP	EC	PA	CA	CG	CT
*A. conyzoides*	22	20	—	18	11	—	—	—	—
*T. erecta*	21	20	—	16	—	13	—	—	—
*C. morifolium*	14	—	—	—	—	—	—	—	—
*T. officinale*	10	—	—	—	—	—	—	—	—
*A. vulgaris*	—	—	—	—	—	—	—	—	—
*C. coronarium*	—	—	—	—	—	—	—	—	—
*H. annuus*	—	—	—	—	—	—	—	—	—
*V. amygdalina*	—	—	—	—	—	—	—	—	—
*W. trilobata*	—	—	—	—	—	—	—	—	—

—: no inhibitory zone.

**Table 4:** IZD (mm) of the fractions determined by well agar diffusion assay.

Plant species	Part tested	Fractions	IZD (mm)				
			SA	SR	KP	EC	PA
*A. conyzoides*	Aerial parts	*n* – hexane	—	—	—	—	—
		CHCl_3_	11	11	—	—	—
		EtOAc	23	21	15	14	—
		EtOH	9	—	10	—	—

*T. erecta*	Flower	*n* – hexane	11	—	11	—	—
		CHCl_3_	11	—	10	—	—
		EtOAc	17	12	18	—	11
		EtOH	—	—	—	—	—

*C. morifolium*	Flower	*n* – hexane	—	—	—	—	—
		CHCl_3_	13	—	—	—	—
		EtOAc	14	—	—	—	—
		EtOH	—	—	—	—	—

*T. officinale*	Aerial parts	*n* – hexane	—	—	—	—	—
		CHCl_3_	—	—	—	—	—
		EtOAc	11	—	—	—	—
		EtOH	—	—	—	—	—

—*:* no inhibitory zone.

**Table 5:** Antimicrobial activity of the essential oils determined by disc diffusion assay.

Essential oils	IZD (mm)								
	SA	SR	EF	KP	EC	PA	CA	CG	CT
*A. conyzoides*	15	—	—	—	—	—	—	8	10
*A. vulgaris*	23	10	—	—	—	—	14	15	16
*H. annuus*	10	—	—	—	—	—	—	—	11

—: no inhibitory zone. MIC values of active extracts and essential oils on standard strains.

**Table 6:** MIC and MBC values (mg/ml) of crude extracts and fractions of four selected plant materials.

Plant species	Part tested	Fractions	MIC/MBC (mg/ml)				
			SA	SR	KP	EC	PA
*A. conyzoides*	Aerial parts	Cru.Ext	2.5/2.5	10/20	5/5	25/25	—
		CHCl_3_	20/40	20/40	—	—	—
		EtOAc	2.5/5	5/10	2.5/2.5	10/10	—
		EtOH	20/20	—	10/20	—	—

*T. erecta*	Flower	Cru.Ext	0.78/1.56	3.13/6.25	1.25/1.25	—	2.5/2.5
		*n* – hexan	1.56/3.12	—	5/5	—	—
		CHCl_3_	1.56/3.12	—	5/5	—	—
		EtOAc	3.13/3.13	6.25/12.5	2.5/2.5	—	5/5

*C. morifolium*	Flower	Cru.Ext	30/30	—	—	—	—
		CHCl_3_	15/30	—	—	—	—
		EtOAc	15/15	—	—	—	—

*T. officinale*	Aerial parts	Cru.Ext	20/20	—	—	—	—
		EtOAc	10/10	—	—	—	—

Cru.Ext: ethanol crude extract; —: no inhibitory zone.

**Table 7:** Determination of minimum inhibitory concentration (MIC) of essential oils.

Essential oils	MIC/MBC (*μ*l/ml)				
	SA	SR	CA	CG	CT
*A. conyzoides*	3.75/7.50	—	—	7.5/15	10/20
*A. vulgaris*	2.50/2.50	6.25/6.25	6.25/12.5	5/10	5/10
*H. annuus*	3.75/7.50	—	—	—	10/20

Antimicrobial activity on uropathogenic strains of *E. coli* and *K. pneumoniae*.

**Table 8:** Antimicrobial activity of the ethyl acetate fraction of *A. conyzoides* determined by agar well diffusion assay.

Strains	IZD (mm)
E3	10.83
E7	11.43
E25	12.17
E36	13.33
E77	13.43
E38	13.67
E27	14.47
E39	14.47
E84	14.97
E68^*∗*^	15.43
E63	20.37
E51	21.40
E42	22.37
E94	22.37
E72	23.27
K18	9.33
K27	10.03
K19	11.23
K20	11.37
K15	11.67
K23	11.83
K14	11.93
K21	12.10
K28	12.37
K25	13.23
K22	13.27
K16	14.20
K29	17.67
K26^*∗*^	19.27
K17	19.73

^*∗*^ESBL-producing strain.

## Data Availability

The data that support the findings of this study are available from the corresponding author upon reasonable request.
